# Factors that influence a patient’s decision to engage in genetic research

**DOI:** 10.3389/fpubh.2023.865786

**Published:** 2023-05-22

**Authors:** Amal Almutairi, Fatimah Abdulrahman Alqubaishi, Ebtehal A. Alsolm, Albandari Binowayn, Rania Almohammad, Tariq Wani, Aljohara Ababtain, Udai Alkadi, May M. Alrashed, Malak Althagafi, Leen Abu-Safieh

**Affiliations:** ^1^King Abdulaziz City for Science and Technology, Riyadh, Saudi Arabia; ^2^Genomics Research Department, Saudi Human Genome Project, King Fahad Medical City, King Abdulaziz City for Science and Technology, Riyadh, Saudi Arabia; ^3^Research Services, Department of Biostatistics, Research Center, King Fahad Medical City, Riyadh, Saudi Arabia; ^4^Department of Clinical Laboratory Sciences, College of Applied Medical Sciences, King Saud University, Riyadh, Saudi Arabia; ^5^Bioinformatics and Computational Biology Department, Research Center, King Fahad Medical City, Riyadh, Saudi Arabia

**Keywords:** genetic research, participation, concerns, survey, attitude

## Abstract

**Introduction:**

The most challenging step in clinical research studies is patient recruitment. Many research studies do not reach their targets because of participant rejection. The purpose of this study was to assess patient as well as the community knowledge, motivation, and barriers to participate in genetic research.

**Methods:**

A cross-section study was conducted between September 2018 and February 2020 using face-to-face interviews with candidate patients from outpatient clinics at King Fahad Medical City (KFMC), Riyadh, Saudi Arabia. Additionally, an online survey was conducted to assess the community’s knowledge, motivation and barriers to participate in genetic research studies.

**Results:**

In total, 470 patients were interviewed for this study, with 341 being successfully recruited for the face to face interview, and the other patients being refused owing to time constraints. The majority percentage of the respondents were females. The respondents’ mean age was 30, and 52.6% reported having a college degree. The survey results from 388 participants illustrated that around 90% of the participants, participated voluntarily due to a good understanding of genetics studies. The majority held positive attitudes toward being part of genetic research, which exceeded the reported motivation score of >75%. The survey indicated that >90% of individuals were willing to participate to acquire therapeutic benefits or to receive continued aftercare. However, 54.6% of survey participants were worried about the side effects and the risks involved in genetic testing. A higher proportion (71.4%) of respondents reported that lack of knowledge about genetic research was one of the barriers to rejecting participation.

**Conclusion:**

Respondents reported relatively high motivation and knowledge for participation in genetic research. However, study participants reported “do not know enough about genetic research” and “lack of time during clinic visit” as a barrier for participation in genetic research.

## Introduction

Genetic research plays an important role in improving the health of individuals and community since it provides preliminary and sometimes confirmed information about individual susceptibility to specific diseases, which is very beneficial to participants. This genetic information has become crucial for the development of diagnostic, preventive, and therapeutic strategies (1). However, genetic research usually shows lower participation rates in comparison to other types of research areas (2).

Patient recruitment for research studies is the most challenging step, and many research studies cannot complete the target sample size because there are not enough subjects willing to participate (1–3). Achieving the final data for evaluation is based on successful patient retention. However, failure in achieving the original target for recruitment has been a common issue in many research studies (4, 5). An insufficient number of samples affects the timeline of projects and has the potential to lead to skewed results (2, 6, 7). Studies can be extended to increase the likelihood to reach the target sample size, however, this will consume more time, effort and create a financial burden on the research team and resources. Achieving an adequate number of participants increases the reliability of data and allows to draw definite conclusions (8, 9). A previous study reported that approximately one-third of trials achieved their sample recruitment target, but more than half of the trials needed to extend their duration to reach their target (1, 3). Another study reported that out of 2,579 eligible trials, 19% were terminated due to the inadequate number of participants, while the remaining completed studies were closed with less than 85% of the expected participation (10).

Effective communication of knowledge related to genetics in clinical research practice is essential to achieve the required target numbers for genetic research projects. Many factors such as knowledge and attitude towards participation in genetic research studies play an important role in successful recruitment (11, 12). Specific to genetics research, previous studies have reported factors such as altruism, and seeking personal health information as some of the common motivating factors for participating in genetic research (13). Meanwhile, commonly reported concerns cited for not participating in genetic research were logistical barriers, and risks related to ethical, legal, or social implications (14).

Thus, a better understanding of the factors related to knowledge, motivation and barriers related to participation will aid in the planning of genetic research projects, estimating a reasonable target, and strategizing the recruitment plan to achieve the target and thus aid in the completion of the study.

Among the Saudi population, measuring knowledge toward clinical research is critical for assessing acceptance and providing a strong support evidence to improve the recruitment process. Several studies were conducted to assess the knowledge of the local Saudi population on this topic (11, 15, 16). However, only one study aimed to assess the level of knowledge with regard to genetic testing among college students in Saudi Arabia (16). As a result, the purpose of this study is to examine the patient’s reasons for accepting or rejecting participation in genetic research. In addition, to capture a range of responses, the study also carried out a survey among the community adults to determine their knowledge, barriers, and motivation to participate in genetic research studies as well as the participant’s expected information before taking part in genetics research projects.

## Materials and methods

### Study design and participants

A cross-sectional study was conducted between September 2018 and February 2020. The study participants included patients from four laboratory-based genetics projects focused on different genetic diseases, and outpatient clinics at King Fahad Medical City (KFMC), Riyadh, Saudi Arabia. The outpatient clinics included ophthalmology clinic, endocrinology clinic, obstetrics and gynecology clinic, and oncology clinic. A face-to-face interview was conducted among the patients.

Moreover, the study also included Saudi adults from the general population. An online survey was conducted among them to assess the community’s knowledge, motivation, and barriers to participate in genetic research studies.

### Recruitment of participants

#### Recruitment of patients from clinics

For the recruitment of patients, the Genetics Research Department recruitment team consisted of seven tained research assistants with similar scientific and training background who identified patients through the clinic schedule and coordinated with the clinical team for the recruitment process using convenience sampling following explanation about genetic research and given examples. A total of 1,400 patients is the target for the four laboratory-based genetics projects. Research assistants approached patients, explained the study, and invited them to take part in the study. A total of 470 patients were approached to participate in the genetics research study and patients that did not agree to participate were asked to provide a reason for their rejection.

#### Recruitment of adults from community

For the recruitment of adults from the Saudi population, the research center database was used. The research center database is a cumulative database of different department activities and research projects. The survey was shared with participants via emails and WhatsApp using their contact info in the database. Moreover, the research team circulated the survey link to their personal contacts as well.

**Figure 1:**
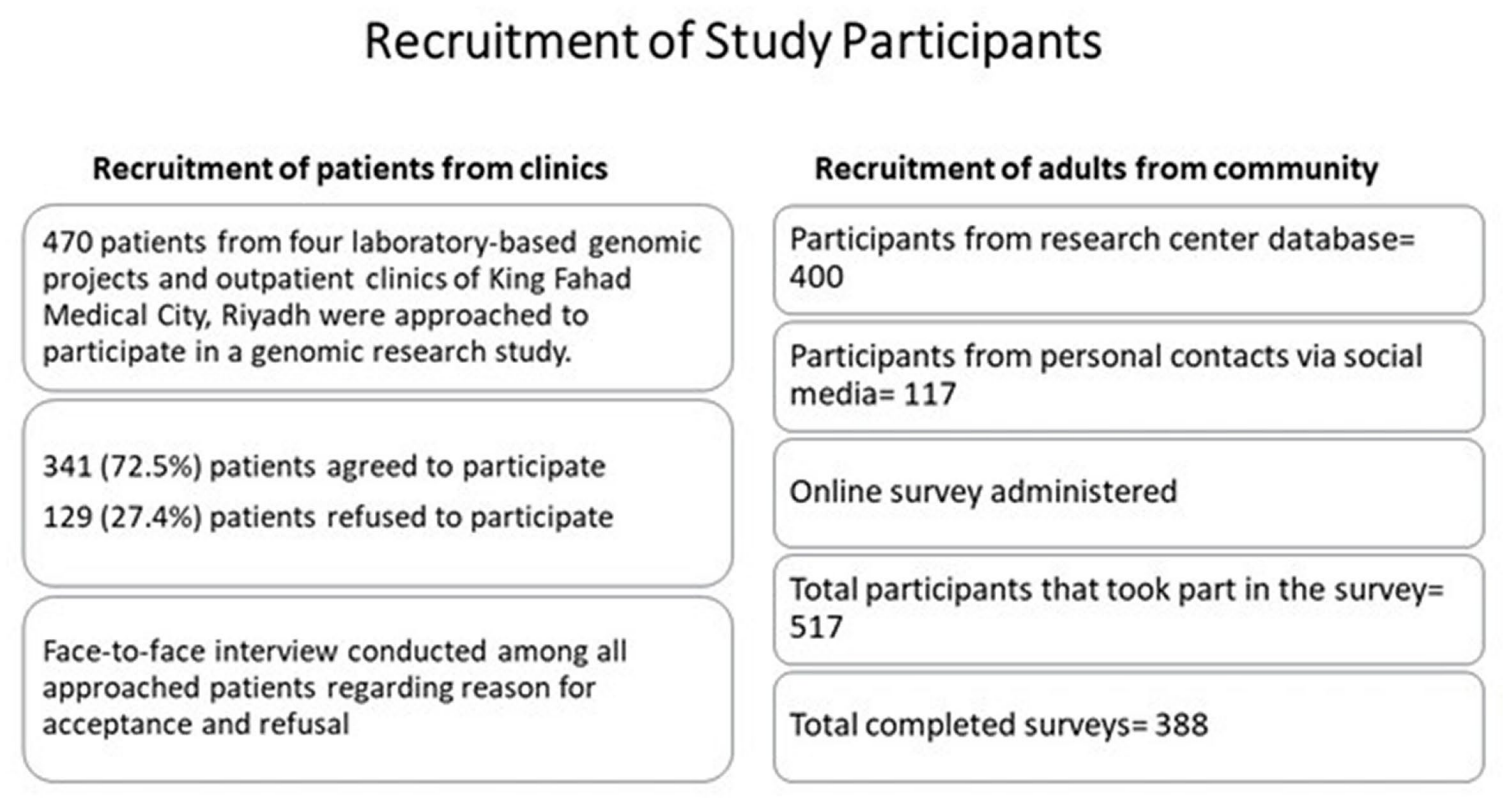
Recruitment of study participants.

A total of 400 participants registered in the database were approached to participate in the online survey. Additional 117 participants were included from the research team’s personal contacts. In total 517 participants filled the survey and 388 completed the survey (see Figure 1).

The online survey was sent randomly to participants from the general population using SurveyMonkey,^1^ which is commonly used for conducting secure online surveys.

Adults and their family members, above 18 years old from both gender who could read Arabic and agreed to participate were included while subjects who do not agree to participate where excluded from this study.

### Data collection tool

#### Questionnaire development

A pre-designed, self-administered questionnaire was used to collect information. The survey questions were designed after an exhaustive literature search (5, 6), and the questionnaire was separated into several sections. A group of experts in research methods and genetic research ensured the questionnaire’s face validity before distributing it to the study population. Some of the question were modified or deleted as per the recommendations of the expert committee since they were either off topic or not suitable. To confirm that the questionnaire was clear and reliable, a pilot was conducted with 50 participants. The reliability was determined using Cronbach’s alpha, which was >0.50.

#### Questionnaire

For the face-to-face interviews with patients, questions related to reasons for accepting or rejecting the participation in genetic research was asked.

For the online survey among the general population, the Arabic language questionnaire was composed of the following sections: personal information which includes name, age, gender, education level, and marital status. Knowledge about genetic research included asking about previous knowledge of genetic research, the benefit of genetic research for patients and community, concerns of confidentiality of the information provided, the role of genetic research in improving medical knowledge and health care and if the genetic research can help in disease management. Motivation was assessed by asking participants for their opinion on therapeutic benefit for the patient or his family, personal interest, recommendation by the patient physician, altruism and influences by family members. The percentage score of knowledge, motivation, barriers was categorized as <50.0, 50.0–75.0, and > 75.0.

Barriers to participate was assessed by asking participants reasons for rejections which included concerns about risks, lack of time during clinic visits, fear of needle, previous bad experience with research and lack of knowledge.

Additionally, we asked the type of information people wanted to know before participation such as side effects, risks, sample collection process, financial incentive and personal benefit.

#### Ethical considerations

The study received approval from the Institutional Review Board Committee of KFMC. A written informed consent was obtained from the patients who agreed to participate in this study. Complete anonymity was maintained to protect participants’ identity and to ensure confidentiality of data.

No personal information was included on the online survey to protect their identity and to ensure the anonymity and confidentiality of the data.

#### Sample size calculation

The sample size was determined with the help of PASS 11.0 software. It assumed 70% patient compliance to participate in the study based on the findings of previous studies (3, 4), who were willing to join genetic projects because of the desire to help others, the Confidence Intervals for One Proportion with the 90% power at 95% confidence interval. The estimated sample size for the present study is *N* = 341. A sample size of 341 produces a two-sided 95% confidence interval with a width equal to 0.10 when the sample proportion is 0.70.

#### Reliability of the questionnaire

The statistical test Chronbach alpha used to measure the internal consistency. It’s the most valuable test for indicating scale for reliability of any given measurements.

Internal consistency of the study items was analysed and the Cronbach’s alpha value for knowledge was 0.501 which indicates low internal consistency. Regarding survey responses about motivation, as measured with nine questions, Cronbach’s alpha value showed high internal consistency (0.724). Participants’ responses regarding barriers against participation showed Cronbach’s alpha equal to 0.724, again showing high internal consistency.

#### Statistical analysis

The socio-demographic variables age, gender, education level, and marital status are presented as categorical data. The 27 response items about the genetic research in the questionnaire are sub-divided into three sections: Knowledge (9 items), Motivation (9 items) and Barriers (9 items). All 27 items followed the Bernoulli process and thereby were measured on a binary scale, such as agreement or disagreement with the respective statement.

SPSS 25.0 software was used for data simulations to attain at least 70% internal consistency within the study sample. Data were described as percentages and the mean cumulative percentage scores. The association between sociodemographic characteristics and the four distinguished dimensions Knowledge, Barriers, Motivation and Overall Assessment percentage scores was measured by Chi square test and Fisher exact test. All the inferences were drawn at 95% confidence interval and value of *p* < 0.05 was considered significant.

## Results

### Results from face-to-face interviews

A total of 470 patients were approached and the number of patients who participated in the current study from is 341 (72.5%), leaving 129 that refused to participate (27.4%) in the study.

Results of the face-to-face interviews with patients regarding the reasons for patients agreeing or disagreeing to join research projects showed that 250 (70%) of participants stated altruism as the main reason for participation, followed by an individual’s interest with (19.2%) and an individual’s personal benefit of 7.3%. With regard to reasons for rejecting participation, 67 (62%) of participants stated lack of time as the main reason for rejection, followed by lack of knowledge (11%). Fear of needles and emotional reasons showed a similar response rate of 10%.

### Results from the online survey

In total 388 participants completed all survey items, out of 517 participants representing a response rate of around 75%.

#### Demographics

Participants were predominantly female (70%), and the mean age of the respondents was 30 years. A college degree was reported by 52.6% of participants. Table 1 shows the complete demographic data collected from the survey.

**Table 1:** Descriptive statistics: percentage score of responses among demographic data and all survey items including knowledge, motivation, and barriers.

Characteristic	Description	*n* (%)
Age	<20	9 (2.3)
	20–40	260 (67.0)
	40–50	74 (19.1)
	>50	45 (11.6)
Gender	Male	114 (29.4)
	Female	273 (70.4)
	Missing	1 (0.3)
Education level	High-school-or-less	62 (16.0)
	University	204 (52.6)
	Postgraduate	120 (30.9)
	Missing	2 (0.5)
Marital status	Single	139 (35.8)
	Married	236 (60.8)
	Other	12 (3.1)
	Missing	1 (0.3)
Knowledge	Genetic research is important for health care improvement	380 (97.9)
	Participants can freely withdraw from genetic research anytime	378 (97.4)
	The information obtained from a genetic research is confidential	377 (97.2)
	Genetic research is beneficial to patients	370 (95.4)
	I will encourage my relatives to take part of genetics research	368 (94.8)
	I have heard previously about genetic research	323 (83.2)
	Genetic research have direct benefits on the community	323 (83.2)
	Results of genetic research can help disease management	313 (80.7)
	Genetic research improve community medical knowledge	285 (73.5)
Barriers	I do not know enough about genetic research	277 (71.4)
	Lack of time during clinic visit	260 (67.0)
	Information provided not clear	243 (62.6)
	I might need to take time off work	214 (55.2)
	I’m worried about the risks	212 (54.6)
	My family and friends would disapprove	145 (37.4)
	Fear of needle	115 (29.6)
	My religious or moral beliefs	99 (25.5)
	Bad personal experience with research	40 (10.3)
Motivation	If other patients get benefits from the result	374 (96.4)
	Therapeutic benefits	373 (96.1)
	Knowing that there would be continued aftercare and follow-up	353 (91.0)
	A personal interest in a particular disease/condition	352 (90.7)
	Recommendation of your own physician	337 (86.9)
	Getting access to the latest treatments for a condition I have	334 (86.1)
	Supporting research into a condition within my family	303 (78.1)
	A positive impact directly on my own health	300 (77.3)
	Influence of family members	295 (76.0)
Expected information	Side effects	321 (82.7)
	Risks	297 (76.5)
	I would like to know my results	259 (66.8)
	Likelihood it would help with my condition	246 (63.4)
	What happens after the recruitment?	245 (63.1)
	The time to know my results	240 (61.9)
	The sample collection process	195 (50.3)
	Level of physical involvement	157 (40.5)
	What financial incentive is available	100 (25.8)
	Nothing—I rather not know	13 (3.4)

Cronbach’s alpha was measured as indicator of internal consistency between items (*n* = 388).

#### Level of knowledge in genetics research

Research participants’ knowledge in genetics research significantly influenced nine questions in the survey. The overall score illustrates that nearly 90% of individuals willing to be involved in genetic research projects thought they had a good understanding of genetics studies (with an answering score above 75%). However, Cronbach’s alpha value was 0.501 which indicates low internal consistency (Table 2). The majority of participants (97.9%) indicated that they had some knowledge about the importance of genetics in health care improvement. Accordingly, over 90% of participants stated they would encourage relatives to take part in genetics research (Table 1).

**Table 2:** Basic data: complete and partial response to the survey as well as the total score of knowledge, barriers, motivation (*n* = 300).

Description		*n* (%)
Complete		388 (74.5)
Incomplete		133 (25.5)
Total		521 (100.0)
Percentage score of knowledge	<50.0	8 (2.1)
	50.0–75.0	28 (7.2)
	>75.0	352 (90.7)
	Cronbach’s alpha	0.501
Percentage score of barriers	<50.0	216 (55.7)
	50.0–75.0	116 (29.9)
	>75.0	56 (14.4)
	Cronbach’s alpha	0.661
Percentage score of motivation	<50.0	23 (5.9)
	50.0–75.0	46 (11.9)
	>75.0	319 (82.2)
	Cronbach’s alpha	0.724
Percentage score of all the 27 factors	<50.0	10 (2.6)
	50.0–75.0	202 (52.1)
	>75.0	176 (45.4)
	Cronbach’s alpha	0.624

#### Motivation

Participants in our study had generally positive attitudes towards taking part in genetic research with 82.2% of participants scoring greater than 75% for motivation (Table 2). When asked about their general interest in genetic research, 90.7% mentioned personal interest in a particular disease as their motivation for participation. In addition, over 90% of the participant were willing to participate if there is a possible therapeutic benefit or they would continue to receive aftercare and follow-up for their condition. Moreover, approximately 90% of respondents indicated that they are willing to participate if they received a recommendation from their physician (Table 1).

#### Barriers

Participants’ responses regarding barriers against participation showed Cronbach’s alpha equal to 0.724, again showing high internal consistency, with 14.4% of respondents scoring above 75% (Table 2).

More than 50% of participants indicated they worried about side effects and risks of genetic testing. A higher proportion 71.4% of respondents reported that lack of knowledge about genetic research was one of the barriers to rejecting participation. Moreover, 63% of respondents indicate a “lack of time during clinic visits” as a barrier to participation (Table 1).

#### Participants’ expected information before participating in genetics research projects

When asked what types of information participants want to know, 83% wanted to know about potential side effects as a result of their participation, followed by 77% interested in risks involved in projects. Over 60% of responses wanted to know the testing results and the likelihood of helping their existing condition. A smaller percentage of participants (50%) were interested to know more about the sample collection process (Table 1).

Table 3 shows the association of age with mean score of barriers, motivation and their overall score. It is clear that participants aged less than 20 years have the highest significant mean score for barriers (5.89 ± 1.96) with *p* value = 0.007. There is no significant difference in mean score of motivation and the overall of mean score (*p* value = 0.365, 0.215, respectively).

**Table 3:** Association of age with mean score of barriers, motivation, and overall score.

Domain	<20 years		20–40 years		40–50 years		>50 years		*p* value
	Mean	SD	Mean	SD	Mean	SD	Mean	SD	
Barriers (out of 9)	5.89	1.96	3.91	2.10	4.53	2.28	4.47	1.91	0.007*
Motivation (out of 9)	7.78	1.20	7.88	1.58	7.47	1.95	7.73	1.81	0.365
Over all for barriers and motivation (out of 18)	13.67	2.74	11.79	2.72	12.00	2.97	12.20	2.69	0.215

*Significant *p* value.

Likert scale was used with two points (No = 0, yes = 1) with a maximum total score of 9 for every domain (Barriers, Motivation) and a maximum score of 18 for overall scores of the two domains.

Table 4 shows the association of gender with mean score of barriers, motivation and their overall score. It is clear that there is no significant difference between male and female with regard to the mean score of barriers, motivation and the overall mean score (*p* value = 0.491, 0.075, 0.585, respectively).

**Table 4:** Association of gender with mean score of barriers, motivation, and overall score.

Domain	Male		Female		*p* value
	Mean	SD	Mean	SD	
Barriers (out of 9)	4.22	2.07	4.10	2.17	0.491
Motivation (out of 9)	7.56	1.85	7.89	1.59	0.075
Over all for barriers and motivation (out of 18)	11.78	2.75	11.99	2.79	0.585

Likert scale was used with two points (No = 0, yes = 1) with a maximum total score of 9 for every domain (Barriers, Motivation) and a maximum score of 18 for over all scores of the two domains.

Table 5 shows the association of education level with mean score of barriers, motivation and overall score. It is clear that participants with educational level high school or less have the highest significant mean score of barriers and the overall score (5.15 ± 2.13, 12.97 ± 2.94, respectively, with *p* value < 0.001). Meanwhile there is no significant difference in mean score of motivation (*p* value = 0.357).

**Table 5:** Association of education level with mean score of barriers, motivation, and overall score.

Domain	High school or less		University		Postgraduate		*p* value
	Mean	SD	Mean	SD	Mean	SD	
Barriers (out of 9)	5.15	2.13	4.28	2.02	3.35	2.08	<0.001*
Motivation (out of 9)	7.82	1.63	7.94	1.52	7.55	1.90	0.357
Over all for barriers and motivation (out of 18)	12.97	2.94	12.23	2.64	10.90	2.63	<0.001*

*Significant *p* value.

Likert scale was used with two points (No = 0, yes = 1) with a maximum total score of 9 for every domain (Barriers, Motivation) and a maximum score of 18 for over all scores of the two domains.

Table 6 shows the association of marital status with mean score of barriers, motivation and their overall score. It is clear that there is no significant difference with respect to marital status in the mean score of barriers, motivation and the overall mean score (*p* value = 0.068, 0.512, 0.231, respectively).

**Table 6:** Association of marital status with mean score of barriers, motivation, and overall score.

Domain	Single		Married		Other		*p* value
	Mean	SD	Mean	SD	Mean	SD	
Barriers (out of 9)	3.99	2.10	4.28	2.16	3.00	2.04	0.068
Motivation (out of 9)	7.95	1.47	7.68	1.81	7.83	1.11	0.512
Over all for barriers and motivation (out of 18)	11.94	2.72	11.96	2.84	10.83	1.95	0.231

Likert scale was used with two points (No = 0, yes = 1) with a maximum total score of 9 for every domain (Barriers, Motivation) and a maximum score of 18 for over all scores of the two domains.

## Discussion

Understanding the factors that affect a patient’s decision to participate in genetics research could help to improve recruitment strategies and overcome barriers.

In our study, over 70% of the participants were willing to participate in genetic projects because of the desire to help others and the results showed that altruism is the predominant reason for patient acceptance to participate; this includes the wish to help other patients or to improve medical knowledge. The concept of altruism as the main contributor for patient participation in genetics research has been discussed previously in many studies (2, 17, 18). Truong et al. reported that out of 253 participants in cancer trials, 120 patients (47%) selected altruistic motivations as the main factor for their participation (18, 19).

With regard to barriers, in the current study 62% patients cited time constraint as a reason for declining to participate in genetics research. Previous studies have also reported time constraints as the main reason for refusing to participate (2, 7). Previous studies further reported the issues related to time commitment as the main reason for refusal (19–22). Thus, giving the patient the choice of setting the appropriate time for them to visit the clinic again and complete the participation process might reduce rejection to participate. Moreover, the most recommended suggestion to minimize barriers is to make the process of recruitment shorter and smoother for participants (9). Furthermore, the type of study and nature of the patient’s disease was also found to influence the decision to participate.

Our results illustrate there was widespread support for genetic studies amongst the participants from the general population, as evidenced by their motivation to participate. Similar support for genetics research has previously been observed in Canada (19) and the USA (19). Our study showed that the study participants were knowledgeable about the importance of genetic research and their rights when they are involved in genetic research as the majority of the study participants reported that genetic research is important for health care improvement, participants can freely withdraw from genetic research anytime, and the information obtained from genetic research is confidential. Although, our study revealed that the majority of the study participants have a good knowledge and had previously heard about genetic research, almost 71% of the study participants reported that they do not know enough about genetic research as a barrier to participation in genetic research.

Furthermore, in the current study, majority of the participants were motivated to participate in research studies if other patients get benefits from the result or they may have a therapeutic benefit from the study. The physician-patient relationship appeared to be a significant factor for acceptance in previous studies (3, 9). In this analysis, it was observed that patients responded better to participation requests following a prior discussion with their physician. In this situation, the patients felt confident about participating in the research and paid less attention to potential side effects, as they depended on and trusted their physician (22). It has been reported previously that patients are more likely to participate if the research idea is introduced by a medical doctor, and especially their own physician (20, 23, 24). Moreover, our results showed that patients are more likely to participate when the side effects and risks are clear.

Previous studies have indicated age, cultural background, and education level as important influencing factors to be considered (6, 8, 9). Accordingly, barriers were significantly associated with younger and less educated participants, in the current study.

We recognize the study limitation that the face to face interview was conducted only in KFMC which may not be reflective of the entire Saudi population. Moreover, the current study did not take into consideration the cultural aspects that may have influenced the response of the particpants.

Based on our study, it is recommended that patients hear about a particular research study from their physician first, after which the recruiter team can provide more details about the research. It is essential that the recruiter considers these attitudes and attempt to enhance the patient’s understanding of the specific research questions. Recruiters should talk slowly and using simple language to meet the understanding level of the patient. These strategies will help patients to better understand the concepts of the research study.

Many participants reported interest in genetic research projects or the process following recruitment. Public support is crucial for research progress and success. Improving community knowledge about genetic research is the primary factor in improving the recruitment process in the future. This report will help establish a foundation and overcome obstacles during the recruitment process for genetic research since genetic diseases incidence and complexity is high in the Saudi population due to consanguinity. These kind of reports are needed to increase awareness and improve genetic research and overcome cultural, social, religious issues related to genetic diseases, such as fear of stigma.

The results presented provide valuable information for clinical genetic research studies to preemptively address concerns of potential participants in genetic research. Increased community knowledge and educatrional activities can be useful to increase awareness and impact of such research on health care. Results of this study can be also used to provide further training for research coordinators and investigation to be considered in future genetic research studies. Qualitative assessments and detailed surveys are needed to provide more comprehensive view about participants attitude towards specific genetic projects.

Future research on this topic with larger groups from different cities, patient free responses assessed *via* qualitative analysis detailed surveys should be conducted with research participants, and the general public to explore their views and understand the unique contextual and cultural factors that play a role in influencing the decision of the local population to participate in genetic research which will help in formulation of strategies that increase recruitment.

## Conclusion

Respondents expressed a high level of motivation and knowledge for participating in genetic research. Participants in the study, on the other hand, reported a “lack of knowledge about genetic research” and “lack of time during clinic visits” as barriers to participation in genetic research.

## Data availability statement

The original contributions presented in the study are included in the article/supplementary material, further inquiries can be directed to the corresponding author.

## Ethics statement

The studies involving human participants were reviewed and approved by King Fahad Medical City IRB office. The patients/participants provided their written informed consent to participate in this study.

## Author contributions

All authors listed have made a substantial, direct, and intellectual contribution to the work and approved it for publication.

## Conflict of interest

The authors declare that the research was conducted in the absence of any commercial or financial relationships that could be construed as a potential conflict of interest.

The reviewer HL declared a shared affiliation with the authors MA, AB, UA, RA, TW, EA to the handling editor at the time of review.

## Publisher’s note

All claims expressed in this article are solely those of the authors and do not necessarily represent those of their affiliated organizations, or those of the publisher, the editors and the reviewers. Any product that may be evaluated in this article, or claim that may be made by its manufacturer, is not guaranteed or endorsed by the publisher.

## Footnotes

^1^https://www.surveymonkey.com
